# Newly reconstructed Arctic surface air temperatures for 1979–2021 with deep learning method

**DOI:** 10.1038/s41597-023-02059-5

**Published:** 2023-03-15

**Authors:** Ziqi Ma, Jianbin Huang, Xiangdong Zhang, Yong Luo, Minghu Ding, Jun Wen, Weixin Jin, Chen Qiao, Yifu Yin

**Affiliations:** 1 Key Laboratory of Plateau Atmosphere and Environment, Chengdu University of Information Technology, Chengdu, Sichuan Province 610225 China; 2 College of Resources and Environment, University of Chinese Academy of Sciences, Beijing, 100190 China; 3 Beijing Yanshan Earth Critical Zone National Research Station, University of Chinese Academy of Sciences, Beijing, 101408 China; 4 NOAA CISESS, North Carolina State University, Asheville, NC 28801 USA; 5 Ministry of Education Key Laboratory for Earth System Modeling, Department of Earth System Science, Tsinghua University, Beijing, 100084 China; 6 Joint Center for Global Change Studies, Beijing, 100875 China; 7 State Key Laboratory of Cryosphere Science, Northwest Institute of Eco-Environment and Resources, Chinese Academy of Sciences, Lanzhou, Gansu 730000 China; 8 Institute of Tibetan Plateau and Polar Meteorology, Chinese Academy of Meteorological Sciences, Beijing, 100081 China; 9 Software Technology Center Asia, Microsoft Corporation, Beijing, 100080 China

**Keywords:** Cryospheric science, Climate sciences

## Abstract

A precise Arctic surface air temperature (SAT) dataset, that is regularly updated, has more complete spatial and temporal coverage, and is based on instrumental observations, is critically important for timely monitoring and improving understanding of the rapid change in the Arctic climate. In this study, a new monthly gridded Arctic SAT dataset dated back to 1979 was reconstructed with a deep learning method by combining surface air temperatures from multiple data sources. The source data include the observations from land station of GHCN (Global Historical Climatology Network), ICOADS (International Comprehensive Ocean-Atmosphere Data Set) over the oceans, drifting ice station of Russian NP (North Pole), and buoys of IABP (International Arctic Buoy Programme). The last two are crucial for improving the representation of the *in-situ* observed temperatures within the Arctic. The newly reconstructed dataset includes monthly Arctic SAT beginning in 1979 and daily Arctic SAT beginning in 2011. This dataset would represent a new improvement in developing observational temperature datasets and can be used for a variety of applications.

## Background & Summary

In recent decades, substantial changes have occurred in the Arctic^1–8^. However, due to the harsh environment, there is currently no complete observation network in the Arctic yet. Prior studies^9–11^ indicated that incomplete coverage of observations in the Arctic may lead to a cold bias in the estimation of the recent Arctic warming, and even underestimate the rate of the recent global warming. Although satellites can provide full coverage observation over the Arctic, they only measure lower tropospheric temperature rather than surface air temperature (SAT)^9,12,13^. Moreover, reanalysis datasets, which are widely utilized in climate science research, also provide full coverage data over the Arctic. However, the reanalysis datasets are not the actual observations^14,15^.

Improving the coverage of Arctic observations has been one of the important focuses of polar research. Martin and Munoz^16^ developed a 6-hour gridded Arctic SATs covering the Arctic ocean and coastal area for 1979–1993 using an optimal interpolation method (OI) based on instrumental observations. The OI analysis was further improved by Rigor *et al*.^17^, and the coverage of the constructed Arctic SAT was expanded to cover the whole Arctic. However, these Arctic SAT datasets have not been updated to represent the dramatic changes that have occurred over the past nearly two decades. Additionally, efforts were also made to increase the observational coverage in the polar regions for the global surface temperature^9,18–25^. A variety of interpolation methods have been used to fill the Arctic’s data gaps with observations from nearby mid-latitudes, however, the resultant Arctic warming may still be underestimated due to a lack of reasonable application of instrumental observations in the Arctic^10^. The global surface temperature dataset NOAA GlobalTemp-Interim^25^ has used the monthly buoy measurements from IABP^17^ (International Arctic Buoy Programme; https://iabp.apl.uw.edu/) to enhance the coverage of observations in the Arctic. However, a large amount of high-temporal resolution temperature records would be lost in the generation of monthly buoy observations due to buoy movement and interruptions of buoy observations.

Deep learning methods have been used in Arctic climate studies, such as sea ice forecasting^26–29^. Recent studies have demonstrated that deep learnings are valuable methods for patching missing data, with partial convolution method outperforming other image inpainting technologies^18,30–33^. Compared to conventional interpolation methods such as kriging and principal component analysis-based infilling, deep learning approaches with partial convolution can produce geographically more realistic temperatures^18^. In this study, we aim to develop a high-quality, regularly updated gridded Arctic SATs since 1979 by employing the deep learning with partial convolution. This new Arctic SAT data will aid in the comprehension of the Arctic climate state, Arctic climate change monitoring, model validation and interaction of Arctic and global climate.

## Methods

### Multi-source observations

This study aims to reconstruct the surface air temperature (SAT) in the Arctic using available instrumental observations as much as possible. Here, SAT was reconstructed based on multi-source daily observations, including SAT at 2 m (SAT at 2 m will be specified in this study, all others represent the surface air temperature) from GHCN-d^34^ (Global Historical Climatology Network-daily) and the Russian NP (North Pole) drifting ice stations^35^, and SAT from the surface marine observations of ICOADS Release 3.0.2^36^ (International Comprehensive Ocean-Atmosphere Data Set, hereafter ICOADS) and the IABP (International Arctic Buoy Programme) buoy observations. In the reconstruction, air temperatures from ICOADS surface marine observations and IABP buoy observations were not corrected to 2 m, but were used directly. The above observational datasets have already undergone quality assurance reviews^34–36^, except for IABP buoy observations. In this work, only IABP buoy observations were subjected to quality control and correction. Moreover, the Arctic is defined as the geographic area north of 60°N, whereas all above-mentioned observations from the Northern Hemisphere were utilized for reconstruction. Due to the application of an equal-area grid in the reconstruction, only the north of 30°N can be fully covered by the reconstructed SATs (more details seen in ‘Base data for reconstruction’ & ‘deep learning model and training’). The expansion of the reconstruction area to lower latitudes enables the inclusion of more climatic interactions in the reconstruction^10^.

GHCN-d^34^ is developed by NOAA (National Oceanic and Atmospheric Administration), collecting near real-time updated measurements of SAT, total daily precipitation and snowfall, etc. from more than 80,000 meteorological land stations in 180 countries and territories worldwide. In 1990, 2010, 2015 and 2020, there were approximately 4000, 5000, 6400, and 6100 terrestrial stations, respectively. During these years, there were 370, 480, 560, and 540 terrestrial stations north of 60°N, respectively.

ICOADS^36^ began in 1662, and is also developed by NOAA by combining observations from multiple sources, such as ships, moored and drifting buoys, coastal stations, and other ocean platforms, etc. ICOADS offers the most extensive surface marine meteorological observations inclusive of gridded SATs. Until the twenty-first century, these observations were sparsely distributed and primarily limited to ice-free regions.

The NP stations^35^ recorded multiple meteorological variables, including SAT and surface temperature (ST), which were obtained from Arctic and Antarctic Research Institute (AARI). NP observations were conducted from 1937 to 1991, interrupted by the collapse of the Soviet Union, and restarted since 2003. Approximately 1–3 NP stations annually report SATs and STs over sea ice in the Arctic, however, STs were not available during summer months (May-September). These NP stations are the manned observing stations and the records are regarded as the most accurate instrumental measurements over sea ice in the Arctic.

**Fig. 1:**
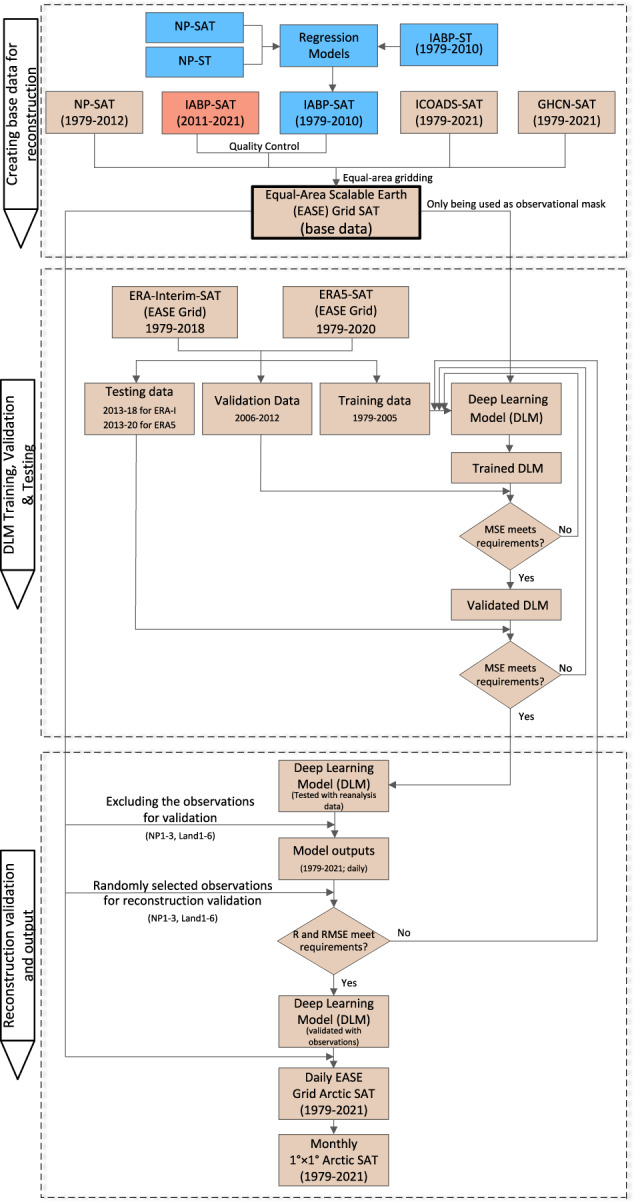
Schematic view of the reconstruction of the monthly Arctic SAT from 1979 to 2021 based on daily observations. The blue boxes illustrate the reconstruction processes for the Arctic SAT from 1979 to 2010. The orange-red boxes indicate the reconstruction processes from 2011 to 2021. The brown boxes depict the share parts of the reconstructions presented above. “R” represents the correlation coefficient. "MSE" and “RMSE” indicate the mean-squared-error and the root-mean-squared-error with units of °C, respectively.

**Fig. 2:**
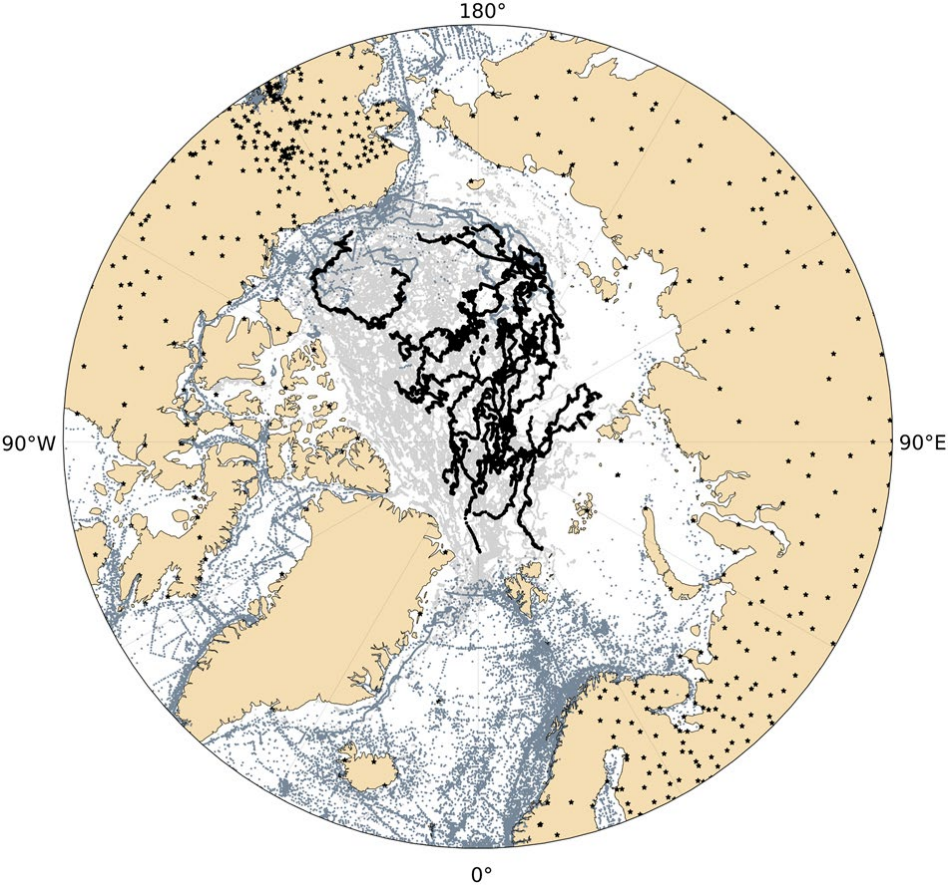
The instrumental observations in the Arctic during 1979-2021. North Pole observations (dark black curves over the ocean) covering 1979-1991 and 2003-2012, IABP buoy observations (grey dots over the ocean), GHCN-d land station observations (black dots on land) covering 1979-2021 and marine observations of ICOADS (dark grey dots over the ocean) covering 1979-2021.

IABP buoy observations began from 1979, which included meteorological variables such as ST, SAT, and surface pressure^17^. Currently, raw records of buoy’s SAT are available from at least 24 buoys annually since 2011, whereas ST records are available from at least 17 buoys annually since 1979. In this study, buoy STs from 1979 to 2010 were first converted to SAT and then used alongside other observations for the reconstruction. Figure 1 describes the schematic overview of the Arctic SAT’s reconstruction. The locations of observations north of 60° N used in the reconstruction are shown in Fig. 2.

### Quality control and correction on buoy observations

Typically, buoy records are available at about fifteen minutes interval (https://iabp.apl.uw.edu). We successively converted these buoy temperature measurements into hourly, 3-hour and 6-hour data using an arithmetic mean. In these steps, a preliminary check of observations was performed. If the temperature exceeds the range of three standard deviations of all records for the same period, the data is discarded as suspicious. Further, the daily observation was produced by averaging the four successive six-hour observations in a day if all four are available; otherwise, the daily observation was set to a missing value. Then, the aforementioned daily observations underwent a screening again to eliminate erroneous observations such as flyers and “flat lined” records and records with obviously unreasonable trajectories.

Daily buoy observations were subjected to quality control and correction using NP data, which are similar to Rigor *et al*.^17^, in the following three steps: 1) Constrain the daily buoy observations with standard deviations of the daily NP observations in the corresponding month. Buoy observations were discarded when σ_a_ < 0.25 σ_NP_ or σ_a_ > 4 σ_NP_ (σ_a_ and σ_NP_ respectively represent the standard deviations of daily buoy and NP observations for a certain month); 2) Remove the outliers. If buoy daily measurements surpass μ_NP_ ± 2 σ_NP_ (μ_NP_ is the monthly mean of NP observations), they are eliminated. 3) Make corrections on summertime (June-July-August) daily buoy observations. The daily buoy observations were filtered using a 1-week moving average. The mean of the filtered buoy observations was replaced by the average of summer NP observations. If the adjusted buoy’s temperature exceeds the NP maximum, they were then changed to the NP maximum. Due to the fact that a considerable volume of heat will be needed to melt sea ice, the buoy summer temperature will be close to the melting point, necessitating this correction^16,17^.

### Conversion of Buoy ST to SAT

Due to unavailability of SAT records prior to 2011 for IABP buoys, there is a potential way by using ST to extend the reconstruction back to 1979. Previous studies^16,37–39^ on the link between temperatures at different altitudes in the Arctic indicated that ST and air temperature over sea ice have a close relationship throughout the year, especially during sea ice melt seasons. As a result, it may be possible to obtain the SAT by exploiting its relationship with ST based on NP data. During the period of May-August, however, ST for NP is unavailable. So, we developed linear regression models for STs and SATs in various temperature intervals based on NP data (Table 1). Using buoy ST and the aforementioned regression models, the SAT (corresponding to ST less than 0°C) prior to 2011 can be generated. This newly produced SATs were then subjected to the same quality control as the original buoy SATs. In addition, when the buoy ST is greater than or equal to 0°C, we approximated the buoy SAT with the buoy ST after removing potentially invalid data. The ST greater than or equal to 0°C is regarded as potentially invalid observation and set as missing value, if the SAT inferred from ST immediately before and after the ST is a missing value. Then, the new SAT including in May-August underwent the same quality control and corrections as the original buoy SAT. Finally, the SATs prior to 2011 are produced (hereafter SAT-n).

**Table 1:** Linear regression relationship between ST and SAT at various ST intervals based on NP data.

Interval	−10 °C~0 °C	−20 °C~−10 °C	−30 °C~−20 °C	<−30 °C
a	0.98	0.99	0.91	0.93
b	−0.66	−0.43	−1.97	−1.46
R^2^	0.91	0.88	0.89	0.93

Here, y = a*x + b, y and x represent SAT and ST, respectively. The ‘a’ is the regression coefficient and ‘b’ is y-intercept.

The conversion of daily ST to daily SAT may introduce errors into the reconstruction of the Arctic SAT. Notably, our purpose is to acquire a set of monthly Arctic SATs for 1979–2021 in this study. Therefore, it is vital to assess the reliability of the daily SAT-n (inferred from the ST) used for the reconstruction of monthly Arctic SAT. We designed three experiments for reconstructing Arctic SAT with the buoy observations for 2011–2020 by using the deep learning model (DLM, further details seen in ‘Deep learning model and training’), when both SAT and ST were available. In the first experiment (expt1), IABP buoy SAT was included in the reconstruction. In the second experiment (expt2), IABP buoy SAT-n (inferred from the ST) was included in the reconstruction, while in the third experiment (expt3), IABP buoy observations (both SAT and SAT-n) were excluded from the reconstruction. The difference between expt1 and expt2 in the reconstructed Arctic SAT can be used to validate the reliability of SAT-n in the reconstruction, whilst the difference between expt1 and expt3 will demonstrate the added value of IABP buoy observations in the reconstruction.

**Fig. 3:**
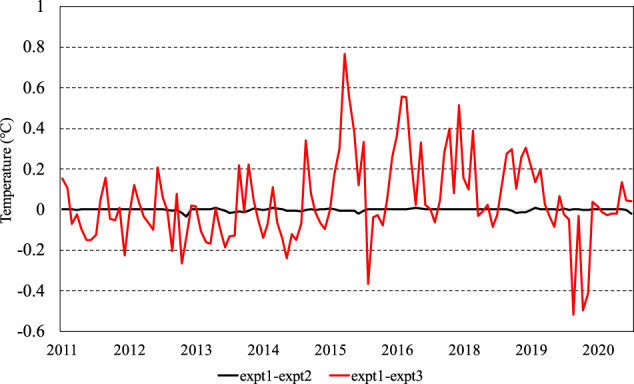
Deviations of monthly average Arctic SAT reconstructed in expt2 and expt3 from expt1, respectively, during 2011-2020. In expt1, buoy SATs were used in the reconstruction; in expt2, buoy SAT-n (SAT inferred from ST) were used in the reconstruction; and in expt3, no buoy observations were used in the reconstruction. The black and red lines, respectively, indicate the expt1-expt2 and expt1-expt3, respectively. The average Arctic SAT is calculated over north of 60°N.

As shown in Fig. 3, there is a subtle difference between expt1 and expt2 regarding the annual average Arctic SAT. And, slightly higher discrepancies (less than 0.1°C) are seen in sea ice melt seasons, which may be due to the direct approximation of the SAT with ST in these seasons. Moreover, these disparities lack discernible linear trend. In addition, the added value (expt1-expt3) resulting from the inclusion of IABP buoy observations in reconstruction is evident and significantly larger than the deviation resulting from substitution of the SAT with SAT-n in the reconstruction. The inclusion of IABP buoy observations results in stronger warming (positive anomaly with a maximum of 0.78°C in expt1-expt3) for the annual average Arctic SAT from the mid-2014 to the early-2019.

**Fig. 4:**
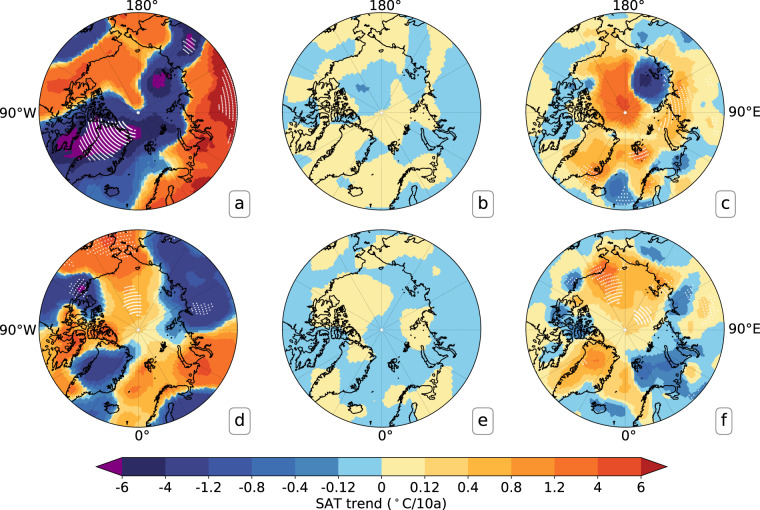
Linear trends of the monthly reconstructed Arctic SAT during 2011–2020. (**a**,**d**) expt1, (**b**,**e**) expt1-expt2, (**c**,**f**) expt1-expt3. (**a**–**c**) represent the SAT’s linear trends in January, and (**d**–**f**) denote the SAT’s linear trends in July. Expt1, expt2 and expt3 are the same as in Fig. 3. The white dots represent the statistical significance at p < 0.05 by using t-test. Note that the contour interval here is designed to be unequal, taking into account the maximum difference between expt1 and expt2.

The differences in the spatial warming trends over the Arctic were also examined for July and January between expt1and expt2, expt1 and expt3, respectively. During 2011–2020, the differences between expt1 and expt2 (Fig. 4b) are considerably smaller than the Arctic warming trends (Fig. 4a) in January. The differences (expt1-expt2) are less than 0.12°C/10a (Fig. 4b) and much smaller than the deviations of expt3 from expt1 (Fig. 4c), which indicate the added value from the inclusion of buoy observations in the reconstruction. It is obvious in Fig. 4c that the largest difference is more than 4°C/10a over the central Arctic Ocean, the Laptev Sea and the East Siberian Sea. In addition, a similar conclusion was also obtained for July. The deviations of expt2 from expt1 (Fig. 4e) are much smaller than the reconstructed SAT trends with inclusion of the IABP SAT (Fig. 4d). They are also significantly smaller than the deviations of expt3 from expt1 (Fig. 4f), especially over the region from the northern Alaska northward to 80°N. These results demonstrate that the buoy ST, after quality control and correction, can be used for the reconstruction of Arctic SAT in the absence of buoy SAT.

### Base data for reconstruction

The locations of buoy, drifting ice station and ship observations move over time. To reconstruct the Arctic SAT, it is necessary to combine all observations using the same geographical grid as a base data. Due to the geographical distortion of the lat-lon grid in the polar region, an equal-area grid (Equal-Area Scalable Earth Grid (EASE-Grid 2.0^40^), hereafter EASE) was used to provide uniform spatial representation for all observations. Similar to prior studies^16,17^, we employed an EASE grid with a cell size of 100 km × 100 km (a total of 32,400 grids with 180 rows and 180 columns). From 2011, there are on average 18 buoy observations on EASE grids every day. In contrast, there are only 3–4 monthly buoy observations (monthly average requiring at least 15 daily observations in one month for the given EASE grid cell). It is clear that more buoy observational information will be retained if the reconstruction is based on daily rather than monthly buoy observations.

After all four source daily observations (quality-controlled SATs from GHCN-d, ICOADS, NP, IABP) were put into the EASE grid cells, the gridded observations were then normalized (to accelerate the convergence speed of model training) as base data using Eq. (1), where X* indicates the temperature after normalization and *X* represents the original data before normalization. *a*, *b* and *t* denote the row and column numbers and the time of the equal-area grid, respectively. The *μ* and *σ* represent the mean and standard deviation of the ERA5 SAT (2 m) over 1979–2020, respectively. As examples, the EASE gridded observations of base data on March 12 and July 20, 2015 were shown in Fig. 6a,d and the observation data gap is obviously seen, although all instrumental observations were integrated into the base data.

1 $$X{\left(a,b,t\right)}^{\ast }=\frac{X\left(a,b,t\right)-\mu \left(a,b\right)}{\sigma \left(a,b\right)}$$

### Training, validation and testing data for DLM

Different training datasets for DLMs may induce disparities in the reconstruction of temperatures over polar areas. Prior studies examined reanalysis datasets, such as MERRA2 (Modern-Era Retrospective analysis for Research and Applications, Version 2)^41^, JRA-55 (Japanese 55-year Reanalysis)^42^, ERA-Interim (ECMWF Re-Analysis-Interim ERA-I)^43^, ERA5 (ECMWF Reanalysis v5)^44^, ASRv2 (Arctic System Reanalysis, Version 2)^45^, etc., in reproducing SAT over the Arctic^46–52^, and indicated that ERA5 and ERA-I are in better agreement with the observed Arctic temperature variations. Wang *et al*.^52^ further indicated that SATs from ERA5 are closer to the Arctic observations when SAT above −25°C, while below −25°C SATs from ERA-I are closer to the observations. So, SATs (2 m) from ERA5 and ERA-I were adopted for DLM training in this study.

Both ERA-I and ERA5 were developed by ECMWF (European Centre for Medium-Range Weather Forecasts) as global reanalyses with a spatial resolution of around 80 km and 31 km, respectively. ERA-I is the third generation of reanalysis accessible from January 1979 to August 2019, which has already been superseded by ERA5. ERA5 is continuously updated and already extended back to 1950^53^. For DLM training and testing, a total of 14,610 daily SAT data from 1979 to 2018 and 15,341 daily SAT samples from 1979 to 2020 were adopted from ERA-I and ERA-5, respectively.

The reanalysis SATs (2 m) from 1979 were divided into a training set of 1979–2005 for model training (19,724 daily samples), a validation set of 2006–2012 for adjusting the model hyperparameters (5,114 daily samples), and a testing set since 2013 for testing model generalisation performance (5,113 daily samples). The binary masks were derived from the base data. During 1979–2020, there was a total of 15,341 binary masks. The 0 indicates the absence of observations in the binary mask, while 1 indicates the presence of observations.

### Deep learning model and training

**Fig. 5:**
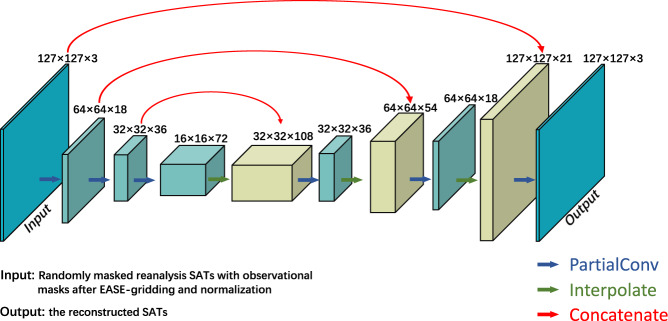
Schematic diagram of structure for the DLM. The left end is the input to the model, while the right end is the output. The numbers on the ‘box’ show the image’s size and number of channels. Blue arrows depict the passage of data through partial convolutional layers to the next layer. The green arrows denote that the data is transferred to the subsequent layer via nearest neighbour up-sampling, gradually restoring the current data to its original size.

This study utilized DLM to fill the instrumental observation gaps in the Arctic. In place of conventional convolutional layers, a UNet-like architecture with partial convolutional layers^54^ was utilized here (Fig. 5 and Table 2). Missing values in the base data are marked with a binary mask that changes with each convolution process. During this procedure, the missing values will be constructed iteratively. As shown in Fig. 5, the model encoding phase is able to extract temperature information across a broad spatial range, allowing the reconstruction to take into account not only nearby but also distant observations. The right side of the diagram illustrates the decoding process. The skip links will establish a connection between the two temperature fields and the two binary masks, transferring the original information to the decoding step and supplying more details for the reconstruction of the missing area.

**Table 2:** Details of network architecture.

Module Name	Filter Size	Channels	Stride	Padding	Nonlinearity
PConv1	7 × 7	18	2	3	ReLU
PConv2	5 × 5	36	2	2	ReLU
PConv3	5 × 5	72	2	2	ReLU
UpSample1	—	72	—	—	—
Concat1	—	72 + 36	—	—	—
PConv4	3 × 3	36	1	1	LeakyReLU
UpSample2	—	36	—	—	—
Concat2	—	36 + 18	—	—	—
PConv5	3 × 3	18	1	1	LeakyReLU
UpSample3	—	18	—	—	—
Concat3	—	18 + 3	—	—	—
PConv6	3 × 3	3	1	1	LeakyReLU

PConv denotes a partial convolutional layer^54^. PConv1–3 are in encoder stage, whereas PConv4–6 are in decoder stage. The skip links are shown via Concat. UpSample is achieved by nearest neighbour interpolation.

The DLM was trained with daily SATs from the reanalysis datasets ERA5 and ERA-I. At first, the reanalysis SATs were put into the same EASE grids as those in base data. The SATs were then normalized based on the monthly mean and monthly standard deviation of ERA5 SATs over 1979–2020. The reconstructions were performed on EASE grids containing 127 rows and 127 columns. The EASE gridded reanalysis SATs were masked with the binary mask before being input into the DLM to reconstruct the corresponding reanalysis SATs. To increase the generic capability, the DLM was trained with the reanalysis SATs by randomly selecting the aforementioned binary masks.

In this study, MSE (Mean squared error) was used as the loss function, which is calculated for SATs across the entire Arctic region between the DLM’s output and the corresponding SATs from the reanalysis. The DLM was trained with 6,000 iterations by applying a batch size of 50. Every 100 iterations, the trained DLM with updated parameters was utilized to reconstruct the Arctic SATs in the training and validation sets, respectively. Then, their MSEs were also calculated. The DLM’s training was not completed until the MSE in the training set no longer declines or continues to decline but it begins to grow in the validation set.

### Testing of the trained DLM

**Fig. 6:**
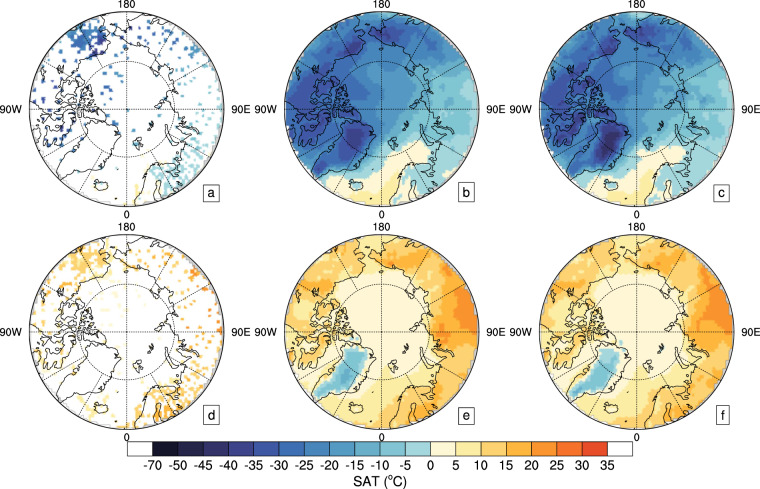
Testing the trained DLM by reconstructing the Arctic SAT on March 12 and July 20, 2015 using the testing dataset. (**a**,**d**) SATs from ERA5 and ERA-Interim (ERA-I) at the observational locations (as input for DLM); (**b**,**e**) the reconstructed SATs with the trained DLM respectively based on ERA5 and ERA-I, respectively. (**c**,**f**) SATs from ERA5 and ERA-I; (**a**–**c**) based on ERA5 on March 12, 2015. (**d**,**e**) based on ERA-I on July 20, 2015.

The ERA5 and ERA-I SATs after 2012 were used as testing set to test the trained DLM by reconstructing Arctic SAT over 2013–2018. Here, the reconstructed Arctic SATs of two days as examples were shown in Fig. 6. The SATs of reanalysis datasets at observational grids that were input to the DLM were demonstrated in Fig. 6a,d. From the observational grids, it is clear that land station observations are mainly located in North America and Europe, with a few coastline observations in Greenland. Buoy observations are concentrated in the western Arctic and a few in the Barents-Kara seas. It is obvious from Fig. 6b,c and Fig. 6e,f that the reconstructed SATs are highly consistent with the reanalysis data, respectively, with spatial correlation coefficients of 0.997 and 0.993. In addition, the reconstructed daily Arctic SATs correlate with that of ERA5 and ERA-I with same temporal field correlation coefficient of 0.997 during the period of 2013–2018. These correlation coefficients are statistically significant. The above test results indicate that the DLM trained with ERA5 and ERA-I is able to reproduce the SATs over the Arctic well based on the limited observations.

## Data Records

The dataset is available at Figshare^55^. As a result of this work, monthly Arctic SAT anomalies relative to 1981–2010 are provided for 1979–2021. The daily Arctic SATs are also presented for 2011–2021. Moreover, the reconstructed SATs north of 30°N are stored in NetCDF format files with 1°x1° latitude-longitude grids, each of which is defined in three dimensions (time, latitude, and longitude). The files ‘*Arctic SAT ano 1* × *1 1979–2021 monthly v1.nc*’ and ‘*Arctic SAT ano 1* × *1 2011–2021 daily v1.nc*’ contain the monthly Arctic SAT anomalies for 1979–2021 and the daily Arctic SATs for 2011–2021, respectively. In these files, “SAT” represents the reconstructed SAT anomalies/SATs, “lat” represents the latitude, and “lon” represents the longitude. Moreover, these datasets can be continuously and consistently updated using the given procedures. Notably, the Arctic SAT reconstruction in this study is based on the SAT over the ocean, land, and sea ice.

## Technical Validation

### Validation of the reconstructed Arctic SAT

Six terrestrial and three marine station observations were randomly chosen to verify the reconstruction. The terrestrial station observations come from GHCN-d and the marine station observations are from NP drifting ice station data. These station observations were excluded from DLM training and reconstruction. The reconstructed SAT, SAT from ERA5 and ERA-I were respectively interpolated to the observational stations over land and the tracks of NP drifting ice stations. Correlation and RMSE (root mean squared error) were adopted to evaluate their relationships with land and marine observations (Table 3).

**Table 3:** Comparison of the reconstructed, ERA-5 and ERA-Interim (ERA-I) Arctic SAT with observations from six terrestrial and three drifting ice stations.

Datasets	Recon		ERA-5		ERA-Interim	
Observation	r	RMSE	r	RMSE	r	RMSE
Land-1 (id RSM00020891, 2011-01~2018-12)	0.996	1.55	0.994	1.79	0.994	1.88
Land-2 (id CA002100402, 2011-01~2018-12)	0.981	3.91	0.986	3.90	0.983	3.68
Land-3 (id USR0000ASNI, 2011-01~2018-12)	0.962	2.66	0.946	3.44	0.935	3.50
Land-4 (id USW00027401, 2011-01~2018-12)	0.985	2.10	0.985	2.43	0.986	2.10
Land-5 (id RSM00023975, 2011-01~2018-12)	0.995	1.58	0.997	1.35	0.996	1.49
Land-6 (id NOE00134886, 2011-01~2018-12)	0.973	1.68	0.969	1.73	0.990	0.894
NP-32 (2003-06~2004-03)	0.994	2.51	0.985	5.80	0.989	3.52
NP-33 (2004-09~2005-08)	0.986	2.46	0.977	5.03	0.979	4.15
NP-34 (2005-09~2006-05)	0.986	2.39	0.963	3.83	0.959	3.93

“r” represents the correlation coefficient. “RMSE” indicates the root-mean-squared-error with units of °C.

Daily observations of six land stations cover 8 years from 2011–2018. The correlation coefficients of the reconstructed SAT with Land-1~Land-6 station observations are 0.996, 0.981, 0.962, 0.985, 0.995 and 0.973, respectively, which are comparable with those from the atmospheric reanalysis datasets (Table 3). All of these correlations were statistically significant. The RMSEs of the reconstructed SAT are at least comparable with those from one of reanalysis datasets. In general, the reconstruction results over land are comparable with those from the reanalysis datasets, and both are close to the observations. This may be due to the assimilation of terrestrial observations in reanalysis datasets.

The three drifting station observations cover the most of the period of 2003–2006 (Table 3), which were also used to evaluate the reconstruction over the Arctic Ocean. As shown in Table 3, the reconstructed SAT is closer to the marine observations than the reanalysis datasets, with higher correlation coefficients and lower RMSE. Much improvement of the reconstructed SAT relative to the reanalysis datasets may be due to the absorption of buoy observations over the Arctic Ocean in the reconstruction, while the reanalysis datasets lack the assimilation of these observations. It is worth noting that (1) this comparison period is before 2011, when buoy’s SAT-n (inferred from buoy’s ST) over the Arctic Ocean is used in the reconstruction; (2) the six terrestrial and three marine station observations were excluded together in the above experiment. Therefore, it can be concluded that our work can reasonably reconstruct the Arctic SATs since 1979.

**Fig. 7:**
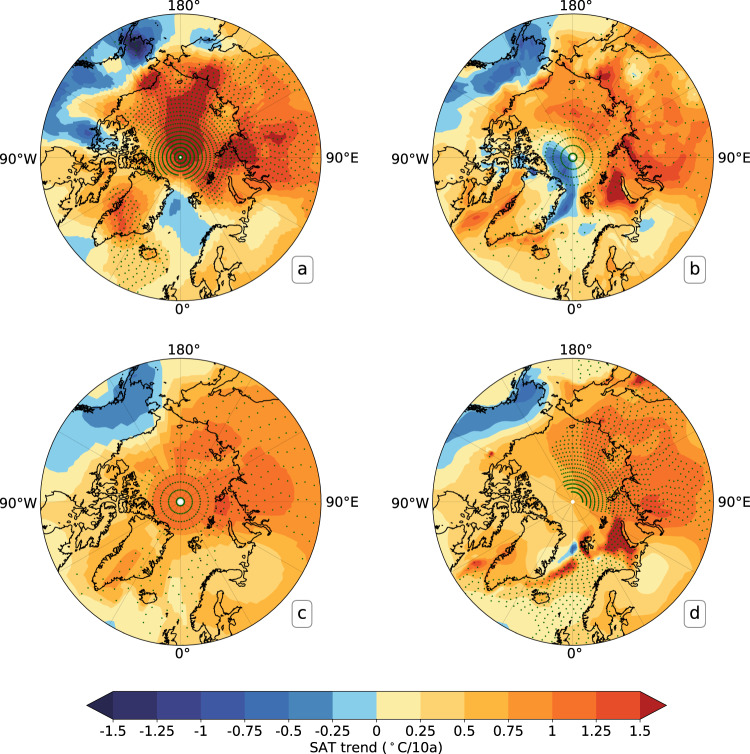
Linear trends of Arctic SATs in March over 1979-2021 for (**a**) the reconstructed Arctic SATs, (**b**) ERA5, (**c**) NASA GISTEMP v4, (**d**) Berkeley Earth. The statistical significance at p < 0.05 is shown with the green cross.

**Fig. 8:**
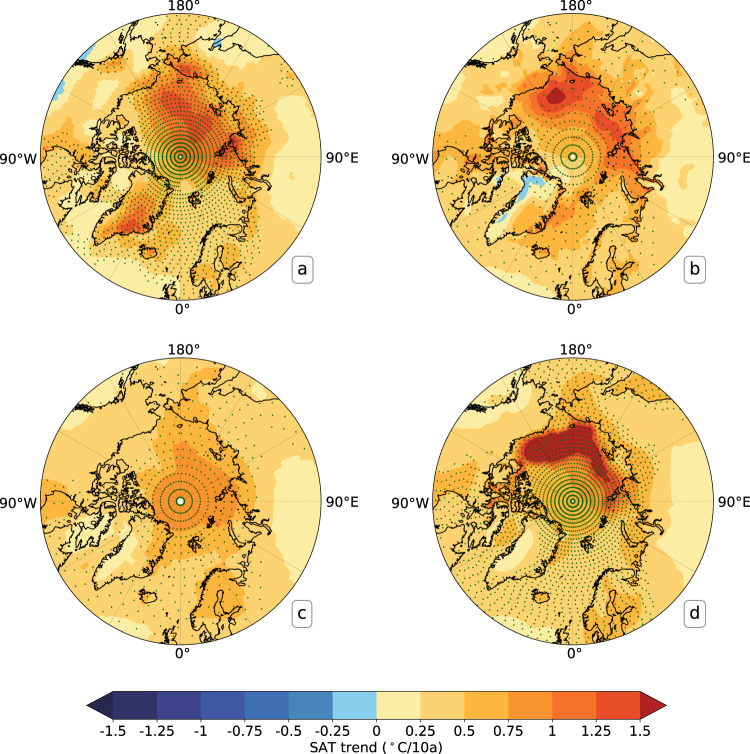
The same as Fig. 7, but for Arctic SAT in September.

In addition, we also investigated the differences in warming trends over 1979–2021 between the reconstructed Arctic SATs and those from ERA5^44^, NASA GISTEMP v4^19^ and Berkeley Earth^21^ in March (Fig. 7) and September (Fig. 8), the maximum and minimum sea ice extent months in the Arctic, respectively. These four data sets consistently demonstrate that March Arctic SATs are warming most significantly over the Arctic Ocean and along the Eurasian coastline (Fig. 7). Nonetheless, the reconstructed SATs indicate a much stronger warming in the Arctic (0.728 ± 0.028°C/10a) relative to ERA5 (0.553 ± 0.025°C/10a), NASA GISTEMP v4 (0.617 ± 0.025°C/10a) and Berkeley Earth (0.632 ± 0.026°C/10a), particularly in the area extending from the central ocean to the East Siberian Sea as well as the coastal region of the northern Alaska, and over Greenland. In September, the Arctic temperature warming trends in the reconstruction (0.55 ± 0.013°C/10a) are weaker than the Berkeley Earth (0.571 ± 0.013°C/10a) and ERA5 (0.571 ± 0.012°C/10a), particularly in the region from the Kara Sea eastward to the Beaufort Sea, but stronger than the GISTEMP (0.463 ± 0.012°C/10a) (Fig. 8). In addition, among the four data sets, the reconstructed SATs for Greenland indicate the strongest warming. All trends above-mentioned were calculated for the area north of 60°N. In general, a warmer Arctic Ocean and Greenland have been reconstructed after additional absorption of observations on sea ice in the Arctic (Figs. 7, 8).

## Data Availability

The code used in this study can be found at 10.6084/m9.figshare.21940490.v1. This code may be updated over time^56^.
